# Development of a QuEChERS–HPLC–FLD Procedure for the Simultaneous Detection of Residues of Florfenicol, Its Metabolite Florfenicol Amine, and Three Fluoroquinolones in Eggs

**DOI:** 10.3390/molecules29010252

**Published:** 2024-01-03

**Authors:** Yawen Guo, Lu Hong, Pengfei Gao, Shuyu Liu, Yali Zhu, Xing Xie, Genxi Zhang, Kaizhou Xie

**Affiliations:** 1 College of Animal Science and Technology, Yangzhou University, Yangzhou 225009, China; dx120200135@yzu.edu.cn (Y.G.); zycx167@163.com (L.H.); mz120211479@stu.yzu.edu.cn (S.L.); mz120211463@stu.yzu.edu.cn (Y.Z.); 2 Joint International Research Laboratory of Agriculture & Agri-Product Safety, Yangzhou University, Yangzhou 225009, China; 3 College of Veterinary Medicine, Yangzhou University, Yangzhou 225009, China; mz120201383@yzu.edu.cn; 4 Key Laboratory of Veterinary Biological Engineering and Technology, Institute of Veterinary Medicine, Jiangsu Academy of Agricultural Sciences, Ministry of Agriculture, Nanjing 210014, China; yzxx1989@163.com

**Keywords:** florfenicol, fluoroquinolones, HPLC–FLD, QuEChERS, egg

## Abstract

A method utilizing high-performance liquid chromatography-fluorescence detection (HPLC–FLD) has been developed and refined for the simultaneous detection of florfenicol (FF) and its metabolite florfenicol amine (FFA) along with three fluoroquinolone (ciprofloxacin (CIP), enrofloxacin (ENR), and sarafloxacin (SAR)) residues in different parts of eggs (whole egg, egg yolk, and egg albumen). The QuEChERS (“Quick, easy, cheap, effective, rugged, and safe”) procedure utilized 0.1 M disodium EDTA solution, water, and acetonitrile as extractants; sodium sulfate, sodium chloride, and trisodium citrate as dehydrating salts; and N-propylethylenediamine and C18 as adsorbents. A dual-channel FLD method was utilized to analyze the target compounds using an XBridge BEH C18 chromatographic column (4.6 mm × 150 mm, 5 μm). The mobile phase was employed isocratically using a solution of 0.01 M sodium dihydrogen phosphate, 0.005 M sodium dodecyl sulfate, and 0.1% triethylamine (pH 4.8) in combination with acetonitrile at a ratio of 65:35 (*V*/*V*). The limits of detection (LOD) and quantification (LOQ) of the analytes ranged from 0.03 to 1.5 µg/kg and from 0.1 to 5.0 µg/kg, respectively. The recoveries of the analytes in the blank egg samples ranged from 71.9% to 94.8% when reference standard concentrations of the LOQ, half of the maximum residual limit (MRL), MRL, and twice the MRL were added. The parameters of the presented protocol were validated and subsequently applied to the analysis of real samples, demonstrating the applicability and reliability of the method.

## 1. Introduction

Eggs are considered a functional food due to their significant production volume, affordability, high nutrient profile, and ease of absorption. Presently, veterinary pharmaceuticals are essential for disease prevention of flocks during rearing process and the treatment of infected individuals under the intensive farming mode of high-density feeding, lack of exercise, excessive growth rate, and poor resistance of the breed. The extensive overuse and indiscriminate misuse of therapeutics results in the accumulation of multiple residues in animals, posing a serious threat to human health due to bioaccumulation effects. For instance, daily intake of ciprofloxacin (CIP) may present an unacceptable health risk in the risk assessment of Ben et al. [1]. Therapeutic residues also present risks associated with carcinogenic, teratogenic, genotoxic, and immunotoxic effects [2]. Furthermore, veterinary therapeutic residues released into the environment, e.g., through animal excreta and pharmaceutical company discharges, continue to be an ecological hazard. The presence of fluorine in fluoroquinolones makes them recalcitrant and less compatible with microbial and enzyme degradation [3]. The continuous accumulation and long-term presence of florfenicol (FF) and fluoroquinolones in ecosystems exerts selective pressure on microorganisms and accelerates the emergence of multidrug-resistant bacteria. Long-term administration and uncontrolled abuse of therapeutics will result in a significant development of resistance in pathogenic microorganisms that were initially susceptible to the therapeutics. The effectiveness of therapeutics diminishes as resistance develops, leading to increasingly severe consequences. Thus, there is a need to establish detection methods based on the maximum residue limits (MRLs) of FF and fluoroquinolone residues in foods of animal origin set by various countries, which can safeguard food health and possible export restrictions.

CIP, which is derived from the metabolism of enrofloxacin (ENR) in animals, can also be utilized as an independent veterinary medication. Florfenicol amine (FFA) is the primary metabolite of FF that exhibits the most prolonged residence in animal liver without any antibacterial activity. In accordance with the newest national standard in China, the MRL of both FF (the sum of FF and FFA) and ENR (the sum of ENR and CIP) is 10 µg/kg in eggs, and that of sarafloxacin (SAR) is 5 µg/kg [4]. While the correlation between the hazard of therapeutic residues and the residue dose remains controversial, establishing a method to simultaneously detect multiple residues is crucial to ensure food safety and monitor the use of therapeutics. The simultaneous detection of FF, FFA, CIP, ENR, and SAR using HPLC–fluorescence detection (FLD) has not been previously reported. Thus, we aimed to develop an HPLC–FLD method for the simultaneous detection of FF, FFA, CIP, ENR, and SAR residues in eggs. Adopting the multiclass, multiresidue method proposed in this study, as opposed to using single-class methods, would considerably enhance laboratory capacity while reducing the costs and time needed to conduct residue analyses.

The QuEChERS (“quick, easy, cheap, effective, rugged, and safe”)-based extraction procedure utilizes liquid–liquid partitioning of acetonitrile or acidified acetonitrile salted out from the aqueous phase by means of salting-out agents such as magnesium sulfate, sodium sulfate, and sodium chloride. Nevertheless, the unmodified QuEChERS-based extraction procedure does not effectively extract polar analytes and metal chelates [5]. In this work, we utilized a modified QuEChERS procedure as the sample pretreatment process for HPLC–FLD assays. In summary, the objective of this study was to construct and validate a repeatable multiresidue QuEChERS–HPLC–FLD method for the simultaneous detection of FF, FFA, CIP, ENR, and SAR residues potentially contaminating eggs for human consumption. The established method is fast and accurate and can be used as a viable alternative to complex single-class, multistep methods, and as a complement to laboratory protocols.

## 2. Results and Discussion

### 2.1. Optimization of Extraction and Cleanup Procedures

The greatest challenge in extracting and purifying multiple therapeutic residues from animal-derived foods lies in the intricate composition of biological components in the sample matrix, encompassing proteins, lipids, and carbohydrates. The high dynamic range, overall chemical complexity, and heterogeneity of compounds within food matrices determine the complexity of extraction and cleanup procedures. Eggs constitute a very complex matrix because of their high lipid and protein content. Some organic extraction solvents may form emulsions and foam with the egg matrix, hindering target compound extraction. Compared with other organic solvents (e.g., methanol), acetonitrile has many advantages, including its characteristics of precipitating proteins and denaturing enzymes [6]. Acetonitrile and water are added to different sample matrices in different proportions to achieve a higher recovery range. The addition of acidified acetonitrile, such as 1% formic acid, enhances the recovery of fluoroquinolones but reduces the recovery of FFA. Additionally, the acid causes the egg to gelatinize, complicating subsequent processing. The addition of EDTA contributed to a more acceptable recovery range for the three fluoroquinolones. We then extracted the sample residues several times with different volume ratios of acetonitrile solutions, and we found that 80% acetonitrile with two more extractions was the most effective after comparison. After one round of defatting, the egg albumen was subjected to nitrogen blowing and resolubilization, completing the LLE process. Both the whole egg and the egg yolk required two rounds of defatting.

The QuEChERS procedure, originally developed for pesticide extraction, is being increasingly introduced in sample pretreatment methods for multiple therapeutic residues [5]. We adhered to the established LLE method and utilized 0.1 M disodium EDTA solution, water, and acetonitrile as extractants to investigate the optimal selection and dose of dehydrating salts and adsorbents in subsequent experiments. The addition of mixed salts not only serves to remove water and salting out but also denatures and disperses the proteins, thus preventing sample clumping and improving extraction efficiency. Based on the sample matrix and analytes in this study, we observed that sodium is more favorable than magnesium sulfate after experimental comparisons. In addition, the combination of sodium sulfate and sodium chloride enhances the transfer of the analytes from the aqueous phase to the organic phase to a greater degree. The dose of the two substances depends primarily on the sample size. To this end, we conducted comparative tests using different combinations: 3 g and 0.5 g of sodium sulfate and sodium chloride, respectively; 3 g and 1 g; 3 g and 1.5 g; 4 g and 0.5 g; 4 g and 1 g; and 4 g and 1.5 g. We investigated the ratio of the two by comparing the recovery of blank whole egg additions. When the doses of sodium sulfate and sodium chloride were 4 g and 1 g, respectively, the lowest values of the mean recovery ranges exhibited a 5% higher difference, as compared to the other ratio combinations. To adjust the pH of the solution, we added trisodium citrate, which contains conjugated acid-base pairs. The inclusion of ceramic homogenizers facilitated the thorough mixing of the solution with the QuEChERS reagents. The N-propylethylenediamine solid-phase adsorbent effectively removes polar interfering substances such as sugars, pigments, fatty acids, and organic acids by hydrogen bonding with the compounds. The C18 adsorbent has a high carbon content and phase coverage and is able to remove some nonpolar impurities and hydrophobic coextracts, such as fats. Comparisons were conducted among different contents of the N-propylethylenediamine and C18 adsorbents: 50 mg and 50 mg, 100 mg and 50 mg, and 100 mg and 100 mg. Optimum purification in the visual was achieved by setting the levels of N-propylethylenediamine and C18 to 50 mg each, respectively. The QuEChERS procedure possesses a few limitations that may impact liquid chromatography–tandem mass spectrometry (LC–MS/MS) detection by causing reduced cleanup and increased susceptibility to matrix interference, but these drawbacks have a comparatively lower impact on LC–FLD detection. Subsequently, n-hexane was employed to remove lipids and potential pigments that could interfere with the detection of residual substances.

A comparative analysis was conducted on three types of filtration membranes: polyvinylidene fluoride, polytetrafluoroethylene, and nylon. It was found that the filtration of nylon membranes interfered with the detection of fluoroquinolones, and the filtering effect of polyvinylidene fluoride surpassed that of polytetrafluoroethylene.

All analytes were spiked into 24 blank egg samples at a concentration of 10 µg/kg. The samples were then pretreated using the established LLE method and QuEChERS procedure, followed by HPLC–FLD analysis. The recovery ranges for the whole egg, egg yolk, and egg albumen achieved with the LLE method were 67.6~89.5%, 68.4~86.1%, and 71.6~90.2%, respectively. The recovery ranges for whole egg, egg yolk, and egg albumen samples achieved with the QuEChERS procedure were 73.2~93.0%, 71.6~92.3%, and 76.1~92.7%. It was evident that the QuEChERS procedure yielded higher recovery ranges than the LLE method. In addition, the QuEChERS procedure is easily standardized and widely adopted, thereby enhancing the speed of sample pretreatment. Thus, the QuEChERS procedure was employed as the sample pretreatment method in this study.

### 2.2. Optimization of HPLC–FLD

The separation performances of the Waters XBridge BEH C18 column (4.6 mm × 150 mm, 5 μm), Waters SunFire C18 column (4.6 mm × 150 mm, 5 μm), PerkinElmer Epic C18 column (4.6 mm × 150 mm, 4 μm), and Dikma Diamonsil Plus C18-A column (4.6 mm × 150 mm, 5 μm) were compared. The SunFire C18 column exhibited poor separation, with peak tailing and a separation resolution for CIP and ENR of less than 1.5. The Epic C18 column failed to clearly separate the FF peak from impurity peaks. Compared to the Diamonsil Plus C18-A column, the XBridge BEH C18 column eluted compounds earlier, yielding sharp peak shapes. Consequently, the XBridge BEH C18 column was selected as the stationary phase for analyte retention and separation.

Various aqueous solutions containing formic acid, phosphoric acid, and citric acid have been tested as mobile phases; however, their application did not meet the experimental requirements. The three fluoroquinolones exhibited well-defined peaks and achieved complete chromatographic separation when acidic mobile phase conditions are utilized. The issue arises from the high polarity of FFA causing early elution under these mobile phase conditions, leading to ineffective separation from impurity peaks. We found that the acidic 0.01 M sodium dihydrogen phosphate solution and acetonitrile, used as components A and B of the mobile phase, respectively, exhibited good separation of each analyte peak. Hydrophobic tail of sodium dodecyl sulfate interacts with the hydroxyl groups on the silica surface of the column, causing the exposure of sulfate anions to the mobile phase. This immobilizes the sulfonic acid groups on the column, introducing a negative charge. Consequently, the column’s capacity to retain positively charged compounds is enhanced. Moreover, sodium dodecyl sulfate forms ion pairs with FFA at a particular pH, resulting in its retention [7]. Sodium dodecyl sulfate can be used to enhance the retention of FFA on chromatographic columns. With an increasing amount of sodium dodecyl sulfate, the retention time of FFA increased. A concentration of 0.05 M sodium dodecyl sulfate enabled an earlier elution of the FFA peak and effective separation from the impurity peak. Triethylamine is commonly employed to neutralize acidity in the mobile phase, effectively reducing the tailing phenomenon observed in chromatographic peaks with alkaline analytes [8]. However, the presence of triethylamine negatively impacted the chromatographic column, and the optimal quantity of triethylamine was determined to be 0.10%. The pH of a 0.01 M sodium dihydrogen phosphate solution, 0.005 M sodium lauryl sulfate, and 0.1% triethylamine was adjusted to 4.8 using phosphoric acid. As a result of this adjustment, the chromatographic peaks of all analytes exhibited no interference or overlap with impurity peaks. Additionally, the peak shape was found to be satisfactory, and the running time was significantly reduced. The present study attempted to use gradient elution protocols to reduce the run time. However, it was discovered that the time needed to restore the initial ratio of the mobile phase to equilibrium did not result in a decrease in the overall run time. Herein, an isocratic elution program was eventually chosen. This study determined the optimal mobile phase conditions to be a solution of sodium dihydrogen phosphate with a concentration of 0.01 M and acetonitrile as components A and B, respectively. Component A contained 0.005 M of sodium dodecyl sulfate, 0.10% triethylamine, and the pH was adjusted to 4.8 using phosphoric acid. The two components were eluted isocratically at a volumetric ratio of 65:35.

**Figure 1:**
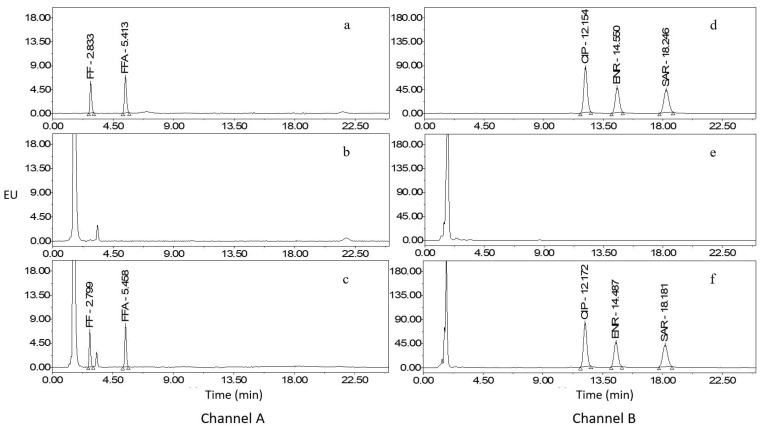
Chromatograms of mixed standard working solution, blank whole egg, and whole egg fortified with standards. Channel A: (**a**), mixed standards (FF: 40.0 µg/L, FFA: 20.0 µg/L); (**b**), blank whole egg; (**c**), whole egg fortified with standards (FF: 20.0 µg/kg, FFA: 10.0 µg/kg). Channel B: (**d**), mixed standards (CIP: 20.0 µg/L, ENR: 5.0 µg/L, and SAR: 20.0 µg/L); (**e**), blank whole egg; (**f**), whole egg fortified with standards (CIP: 10.0 µg/kg, ENR: 2.5 µg/kg, and SAR: 10.0 µg/kg).

**Figure 2:**
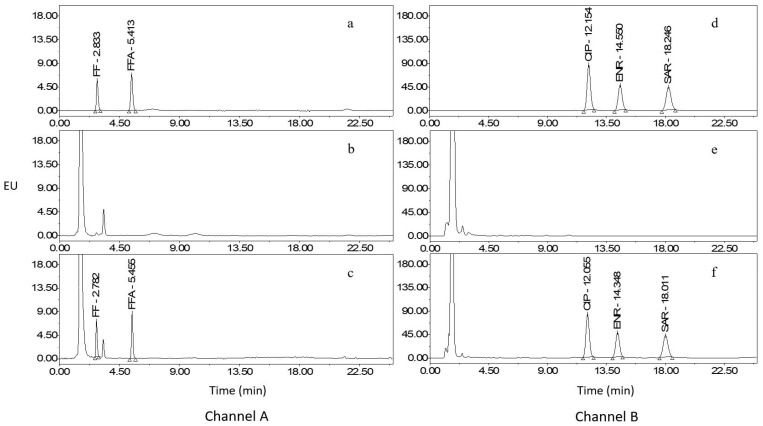
Chromatograms of mixed standard working solution, blank egg yolk, and egg yolk fortified with standards. Channel A: (**a**), mixed standards (FF: 40.0 µg/L, FFA: 20.0 µg/L); (**b**), blank egg yolk; (**c**), egg yolk fortified with standards (FF: 20.0 µg/kg, FFA: 10.0 µg/kg). Channel B: (**d**), mixed standards (CIP: 20.0 µg/L, ENR: 5.0 µg/L, and SAR: 20.0 µg/L); (**e**), blank egg yolk; (**f**), egg yolk fortified with standards (CIP: 10.0 µg/kg, ENR: 2.5 µg/kg, and SAR: 10.0 µg/kg).

**Figure 3:**
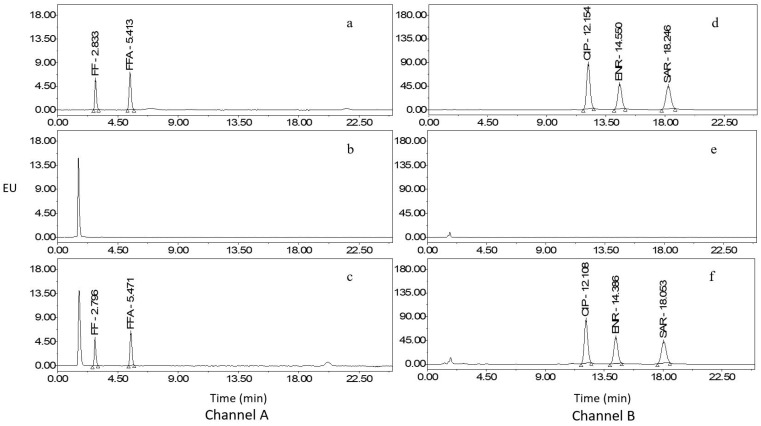
Chromatograms of mixed standard working solution, blank egg albumen, and egg albumen fortified with standards. Channel A: (**a**), mixed standards (FF: 40.0 µg/L, FFA: 20.0 µg/L); (**b**), blank egg albumen; (**c**), egg albumen fortified with standards (FF: 20.0 µg/kg, FFA: 10.0 µg/kg). Channel B: (**d**), mixed standards (CIP: 20.0 µg/L, ENR: 5.0 µg/L, and SAR: 20.0 µg/L); (**e**), blank egg albumen; (**f**), egg albumen fortified with standards (CIP: 10.0 µg/kg, ENR: 2.5 µg/kg, and SAR: 10.0 µg/kg).

The intensity of emitted fluorescence varies among different compound molecules when subjected to the same energy. Thus, it is imperative to determine the optimal detection wavelength for the analyte to maximize fluorescence intensity and enhance method accuracy. In this research, the 3D mode of the fluorescence detector was used to set an emission wavelength, and the optimal excitation wavelength was determined by selecting the wavelength with the highest fluorescence intensity in the excitation spectrum. Subsequently, the selected excitation wavelength was utilized to scan the emission spectrum to identify the wavelength with the highest fluorescence intensity, which was then designated the optimal emission wavelength. We have also employed such a dual-channel detection mode in our previous research [9]. Considering the fluorescence intensity of the analyte within the identical channel, an excitation wavelength of 228.0 nm and an emission wavelength of 279.0 nm were chosen for FF and FFA, and an excitation wavelength of 272.0 nm and an emission wavelength of 450.0 nm were determined for CIP, ENR, and SAR. Chromatograms of mixed standard working solution, blank sample, and blank sample fortified with standards are presented in Figure 1, Figure 2 and Figure 3.

### 2.3. Method Validation

The constructed curves presented a good linear response in the regression analysis of the ratio of peak area to concentration (R^2^ ≥ 0.9998), as detailed in Table 1. The LOD range for the five analytes was 0.03~1.5 µg/kg, while the LOQ range was 0.1~5.0 µg/kg. The accuracy of the established method was also determined for samples fortified with veterinary therapeutic at LOQ, 0.5× MRL, MRL, and 2× MRL concentration levels (listed in Table 2, and detailed data are provided in the Table S1). Good recoveries of all five analytes were obtained, ranging from 71.9% to 94.8%. The between-day (≤6.6%) and within-day precisions (≤7.3%) demonstrated in Table 2 are acceptable. Consequently, the data regarding the method’s linearity, sensitivity, accuracy, and precision were effectively validated to align with the criteria outlined in the guidelines [10,11].

No statistically significant changes were observed in the standard stock solutions and working solutions, which were stored at −80 °C for 3 months (*n* = 24) and 4 °C for 7 days (*n* = 24), respectively. The peak areas of each analyte in the fortified sample matrix extract stored at 4 °C for 12 h did not exhibit any significant changes (*n* = 30). No significant change in the detection peak area was revealed in the peak area of each analyte in the fortified sample matrix extract after subjecting it to three repeated freeze-thaw cycles (*n* = 18).

### 2.4. Comparison with Reported Methods

To date, in the field of chromatography and mass spectrometry techniques, hybrid chromatography and mass spectrometry techniques are frequently employed to simultaneously detect the residues of FF, its metabolite FFA, and fluoroquinolones in animal-derived foods [2,9,12,13,14,15,16,17]. The development and application of high-resolution mass spectrometry techniques have significantly expanded the range of detectable analytes [14,15,16,17,18]. The sensitivity of the LC–MS/MS method may not necessarily be lower than that of the LC method we have established [14,15]. In the detection of FF and fluoroquinolone residues, the majority of studies have primarily focused on a single class of compounds when employing LC methods [19,20,21,22,23,24,25,26,27]. FLD is commonly utilized in LC methods for the detection of FF and FFA [19,20,21]. Additionally, an ultraviolet detector (UVD) and diode array detector (DAD) are also employed in combination with LC methods for the analysis of FF and FFA [22,23]. FLD and UVD have also been utilized for the detection of fluoroquinolones [24,25,26,27]. Among them, Marazuela et al. conducted a simultaneous investigation on both the FLD and UVD methods [27]. Only one study has reported the concurrent detection of FF (including FFA) and fluoroquinolones using LC methods. Barani et al. presented an HPLC–UVD approach to detect FF, FFA, CIP, and ENR in rainbow trout muscle [28]. To our knowledge, no study has yet investigated the simultaneous detection of FF, FFA, and fluoroquinolones using the LC–FLD method. We employed the dual-channel mode of FLD to detect these two types of pharmaceuticals: one channel for FF and FFA and another channel for CIP, ENR, and SAR. This dual-channel mode has been validated in our previous research [9], which ensures the simultaneous detection of both types of pharmaceuticals and avoids response interference between them. The detection method and sample pretreatment method are innovative, efficient, sensitive, and accurate (Table 3). The QuEChERS–HPLC–FLD method we have established provides technical support and a new monitoring scheme for the simultaneous detection of these therapeutic residues in animal-derived food.

### 2.5. Analysis of Real Samples

Forty-two eggs were purchased from various sources, including wholesale markets, supermarkets, retail stores, and e-commerce platforms. These eggs were selected as real samples for this study, deriving from seven different commercial brands: two free-range egg brands and five feedlot industrial egg brands. Each sample was assigned a unique identifier ranging from sample 1 to sample 42. The results are consolidated in Table 4, and the presence of FF, FFA, CIP, ENR, and SAR was not detected.

## 3. Materials and Methods

### 3.1. Reagents, Materials and Solutions

The FF reference standard (CAS No. 73231-34-2, purity ≥ 98.0%) and FFA reference standard (CAS No. 76639-93-5, purity ≥ 98.0%) were obtained from Anpel Laboratory Technologies Inc. (Shanghai, China). The CIP reference standard (CAS No. 85721-33-1, purity ≥ 98.0%), ENR reference standard (CAS No. 93106-60-6, purity ≥ 98.0%) and SAR reference standard (CAS No. 91296-87-6, purity ≥ 98.0%) were purchased from Macklin Biochemical Technology Co., Ltd. (Shanghai, China). Acetonitrile and methanol were of chromatography grade and were provided by Tedia Company Inc. (Cincinnati, OH, USA). Sodium hydroxide, anhydrous sodium dihydrogen phosphate, sodium dodecyl sulfate, triethylamine, and phosphoric acid were of chromatography grade and were acquired from Sinopharm Chemical Reagent Co., Ltd. (Shanghai, China). Disodium ethylenediaminetetraacetic acid (EDTA), sodium sulfate, sodium chloride and trisodium citrate were of analytical grade and were supplied by Sinopharm Chemical Reagent Co., Ltd. (Shanghai, China). N-propylethylenediamine (CAS No. 111-39-7) and C18 (CAS No. 112-61-8), acquired from Anpel Laboratory Technologies Inc. (Shanghai, China), were both analytical-grade products. The purified water (18.25 MΩ.cm, 25 °C) used in the experiment was prepared by a Milli−Q HR 7000 automatic sterilizer and purifier (Merck Drugs & Biotechnology Co., Inc., Fairfield, OH, USA). Polyvinylidene fluoride syringe filters (13 mm × 0.45 μm) were obtained from Thermo Fisher Corporation (Waltham, MA, USA).

FF was dissolved in acetonitrile to achieve a concentration of 1 mg/mL. FFA was dissolved in 1 mL of purified water, and acetonitrile was used to adjust the concentration to 1 mg/mL. CIP, ENR, and SAR were dissolved in 1 mL of a 0.03 M sodium hydroxide solution and brought to a final volume of 10 mL with methanol. These solutions were sealed and stored at −80 °C and stabilized over 3 months. The standard working solution was prepared by serially diluting the standard stock solution with acetonitrile. These solutions were sealed at 4 °C for stable storage over 7 days.

Anhydrous sodium dihydrogen phosphate (1.20 g) and sodium dodecyl sulfate (1.44 g) were accurately weighed and dissolved in purified water to 1000 mL. Then, 1 mL of triethylamine was added, the pH of the solution was adjusted to 4.8 using phosphoric acid, and the solution was used as the mobile phase component A after degassing with an ultrasonic cleaner (P300H, Elma Electronic Ltd., Munich, Germany).

### 3.2. Instrumental Conditions

HPLC analysis was performed using an Alliance e2695 HPLC system (Waters Corp., Milford, MA, USA) coupled to a 2475 fluorescence detector (Waters Corp., Milford, MA, USA).

The vials were maintained in a sample manager at 15 (±5) °C until a 100-μL aliquot was injected into an autosampler, after which it was injected onto an XBridge BEH C18 reversed-phase column (150 mm × 4.6 mm, length × internal diameter, 5 μm) thermostated at 30 °C. A binary mobile phase was used, at a flow of 1 mL/min. Mobile phase component A was 0.01 M sodium dihydrogen phosphate solution (phosphoric acid was used to adjust the pH to 4.8) containing 0.005 M sodium dodecyl sulfate and 0.1% triethylamine, and component B was acetonitrile. Isocratic elution was carried out with mobile phases A and B at a ratio of 65:35 (*V*/*V*).

The excitation and emission wavelengths of channel A for fluorescence detection were set to 228.0 nm and 279.0 nm, respectively. Channel B was operated at excitation and emission wavelengths of 272.0 nm and 450.0 nm, respectively, for the detection of fluoroquinolones.

### 3.3. Extraction and Cleanup Procedures

#### 3.3.1. QuEChERS Procedure

Incurred eggs were obtained from laying hens fed a commercial standard diet and bred at an experimental farm of Yangzhou University (Yangzhou, China). Forty-five laying hens were divided into three groups of 15 hens each and were not given any medication during the feeding period. The Animal Care and Use Committee of Yangzhou University (license number: SYXK(SU) 2021–0044) authorized all procedures. To ensure that the selected portions were representative of the whole, samples were homogenized at 500 rpm for 30 s by using a GD16-96 grinding mill (Xinrui Technology Co., Ltd., Shenzhen, China).

The QuEChERS procedure implemented in this study was built upon the research conducted by Fedeniuk et al. [5]. An aliquot of 2 g of homogenized egg was accurately weighed and then homogenized with 0.4 mL of 0.1 M disodium EDTA solution. The mixture was rotary shaken for 1 min, and then a certain volume (0.1 mL for whole egg, 0.6 mL for egg yolk, and 0 mL for egg albumen) of purified water and 7 mL of acetonitrile were added and shaken in a rotary shaker for 10 min. Then, a mixture of 4 g of sodium sulfate, 1 g of sodium chloride, 0.5 g of trisodium citrate, and ceramic homogenizers was added, the mixture was rotary shaken for 5 min and centrifuged at 8000 rpm for 10 min at 4 °C, and the organic layer was transferred to another centrifuge tube. Fifty milligrams of N-propylethylenediamine and 50 mg of C18 solid-phase adsorbents were added, followed by shaking and centrifugation under the same conditions as in the previous step. Five milliliters of n-hexane was added to the supernatant and subjected to vortexing for 5 min for degreasing. After standing for 5 min, the upper layer was discarded, and the lower extract was evaporated under nitrogen at 40 °C (N–EVAP–24, Organomation Associates Inc., Berlin, MA, USA). The residue was reconstituted with mobile phase component A/acetonitrile at a volume ratio of 65:35, rotary shaken for 3 min, and centrifuged at 8000 rpm for 5 min at 4 °C. The upper layer was filtered through 0.45 μm polyvinylidene fluoride syringe filters and pipetted into the injection vial for immediate HPLC–FLD analysis.

#### 3.3.2. Liquid–Liquid Extraction

For liquid–liquid extraction (LLE), an aliquot of 2 g of homogenized egg was accurately weighed and then homogenized with 0.4 mL of 0.1 M disodium EDTA solution and a certain volume (0.1 mL for whole egg, 0.6 mL for egg yolk, and 0 mL for egg albumen) of purified water. A volume of 7 mL of acetonitrile was added and shaken in a rotary shaker for 10 min. The upper supernatant obtained after centrifugation at 8000 rpm for 10 min at 4 °C was transferred to another polypropylene tube. Ten milliliters of 80% acetonitrile solution was added to the remaining sample residue and rotary shaken for 10 min. The supernatant was transferred, and the remaining residue was extracted again with 80% acetonitrile. The supernatant obtained from a total of three extractions was concentrated to 10 mL in an evaporator (N_2_, 40 °C). Afterwards, 5 mL of n-hexane was added for degreasing, and the mixture was rotary shaken for 5 min before standing for 5 min. The upper n-hexane layer was discarded, and the whole egg and egg yolk samples were subjected to a repeated degreasing process. The lower solution was concentrated to dryness in a centrifugal concentrator (ScanSpeed Vac40, LaboGene Co., Ltd., Helsinge, Denmark). The concentration process of the centrifugal concentrator caused the residue to adhere to a higher position within the wall of the tube. Therefore, 0.5 mL of mobile phase component A/acetonitrile at a ratio of 65:35 (*V*/*V*) was added twice and then thoroughly vortexed for 3 min each time to resolubilize the residue. The clear supernatant obtained after centrifugation at 8000 r/min for 5 min at 4 °C was filtered using 0.45 μm polyvinylidene fluoride syringe filters and then transferred into the injection vial.

### 3.4. Method Validation

The method validation procedure is primarily based on the guidelines issued by SANTE/11813/2017 [10] and FDA Bioanalytical Method Validation 05/24/18 [11] and was designed to evaluate sensitivity (limit of detection (LOD) and limit of quantification (LOQ)), linearity, accuracy, precision, and stability.

The LOD and LOQ values were evaluated based on signal-to-noise ratios of approximately 3:1 and 10:1, respectively. The corresponding series of concentrations for each analyte were fortified and analyzed, the solvent standard curve was obtained by fitting the response to the fortified concentrations, and the linearity of the method was assessed by examining the determination coefficient (R^2^). Six fortified samples were prepared at each of the four concentration levels (LOQ, 0.5× MRL, MRL, and 2× MRL), and the detected concentration was calculated using the solvent standard curve. The recovery, which represents the ratio between the detected concentration and the theoretical concentration, was used to evaluate the accuracy of the method. The LOQ value for FF was equivalent to its 0.5× MRL value, and the fortified level for FF comprised three concentrations. The between-day precision and within-day precision are expressed as the relative standard deviation (RSD) of the recovery. The distinction between the two is that the former is subjected to testing using a single standard curve three times within a day, while the latter is subjected to testing using three standard curves over three consecutive days.

The stock solution, which was stored at −80 °C, underwent analysis after dilution on Days 0, 10, 30, and 90. The working solution, stored at 4 °C, was analyzed on Days 0, 1, 3 and 7. Blank whole egg, egg yolk, and egg albumen samples were subjected to the established sample pretreatment method to obtain blank matrix extracts. Subsequently, the extracts of each sample were mixed and equally divided into six aliquots. The corresponding LOQ levels of each analyte were then added to the aliquots. The fortified extracts were analyzed at different time intervals (0 h, 1 h, 3 h, 6 h, and 12 h) to observe changes in peak area. The corresponding LOQs of each analyte were added to the blank matrix extracts and stored at −20 °C. Eighteen aliquots of each fortified sample matrix extract were removed from storage at −20 °C on Day 1 and thawed at room temperature for a period of 4 h, of which 6 aliquots were analyzed. Another 12 aliquots were refrozen at −20 °C, removed on Day 2 and thawed at room temperature for 4 h, of which 6 aliquots were analyzed. The remaining 6 aliquots were refrozen at −20 °C and subsequently thawed at room temperature for 4 h on Day 3 and analyzed.

## 4. Conclusions

Although multiresidue methods are designed to detect a wide range of analytes, the simultaneous detection of residues from different chemical classes in the same sample using LC methods is not commonly observed. This method demonstrates the successful application of a specific and sensitive QuEChERS–HPLC–FLD method with dual-channels to the analysis of FF, FFA, CIP, ENR, and SAR residues in eggs. The therapeutic residues were extracted from the samples using the QuEChERS procedure, the recoveries of the analytes exceeded 71.9%, and the chromatographic peaks for the analytes were well separated and symmetrical. The method provided satisfactory analytical performance parameters for target analytes, including validated and superior selectivity, sensitivity, linearity, and precision. The proposed method provides technical support for the response to food safety issues and evaluation of the potential risk of therapeutic residues. A more comprehensive matrix/species validation for real sample analysis should be performed in future studies for optimization and enhancement of the method.

## Figures and Tables

**Table 1:** LODs (*n* = 20), LOQs (*n* = 20), linearity ranges, regression equations, and R^2^ values achieved for the detection of analytes using the proposed method.

Analyte	LOD (µg/kg)	LOQ (µg/kg)	Linearity Range (µg/L)	Linearity	R^2^
FF	1.5	5.0	10.0~320.0	y = 6591.3x − 44,059	0.9998
FFA	0.5	2.0	4.0~160.0	y = 16,409x − 53,809	0.9999
CIP	0.05	0.1	0.2~80.0	y = 333,491x − 42,526	0.9999
ENR	0.03	0.1	0.2~20.0	y = 475,677x + 21,687	0.9999
SAR	0.1	0.2	0.4~80.0	y = 151,149x + 2720.6	0.9998

**Table 2:** The recovery (mean (%), *n* = 6 for each level) ranges, along with the ranges of between-day (*n* = 6 for each level) and within-day repeatability (*n* = 18 for each level) expressed as RSD (%).

Matrix	Analyte	Fortified Level (µg/kg)	Recovery	Between-Day RSD	Within-Day RSD
Whole egg	FF	5.0~20.0	83.9~94.6	3.4~4.1	3.4~4.5
	FFA	2.0~20.0	71.9~82.4	3.1~4.6	3.6~5.1
	CIP	0.1~20.0	76.0~84.1	3.5~5.1	3.6~6.1
	ENR	0.1~20.0	87.1~94.8	4.0~5.1	3.9~6.2
	SAR	0.2~10.0	82.0~87.5	3.3~4.9	4.1~5.2
Egg yolk	FF	5.0~20.0	80.4~90.5	3.6~4.6	3.7~5.3
	FFA	2.0~20.0	73.2~81.9	3.9~4.7	3.9~4.9
	CIP	0.1~20.0	72.6~83.8	3.9~4.6	3.7~5.8
	ENR	0.1~20.0	86.2~94.4	3.9~6.6	4.4~7.3
	SAR	0.2~10.0	73.5~80.7	3.5~5.1	3.9~5.1
Egg albumen	FF	5.0~20.0	87.4~92.2	3.9~4.5	4.1~5.2
	FFA	2.0~20.0	75.1~81.5	3.1~4.9	3.4~5.8
	CIP	0.1~20.0	75.3~83.7	3.4~4.5	4.6~5.2
	ENR	0.1~20.0	86.7~94.6	3.9~6.3	4.6~6.4
	SAR	0.2~10.0	82.4~89.3	3.6~6.1	4.2~7.0

**Table 3:** Comparison between the method developed in this study and other LC methods and LC–MS/MS methods for the simultaneous detection of FF, FFA, CIP, ENR, and SAR in animal-derived food samples.

Matrix	Analyte	Detection Method	Sample Pretreatment	LOQ (µg/kg)	Recovery (%)
Egg [2]	FF, FFA, CIP, ENR, SAR, and other analytes	HPLC–MS/MS	Extraction with 0.1% formic acid in acetonitrile: water (8:2, *V*/*V*) and 0.1 M EDTA	-	75.0~108.0
Shrimp [12]	FF, ENR, SAR, and other analytes	HPLC–MS/MS	Extraction with *N*, *N*, *N′*, *N′*-tetramethyl-1,4-phenylenediamine dihydrochloride and acetonitrile	FF: 0.25 µg/L ENR and SAR: 1.0 µg/L	FF: 88.67~92.35 ENR and SAR: 75.21~103.31
Fish [13]	FF, CIP, ENR, SAR, and other analytes	HPLC–MS/MS	Extraction with 0.4% hydrochloric acid in acetonitrile and ethyl acetate, SPE clean-up with Cleanert Alumina N column (500 mg) and HLB cartridge	0.3~1.0	67.7~112.8
Aquaculture products [14]	FF, FFA, CIP, ENR, SAR, and other analytes	UHPLC–MS/MS	Extraction with 0.1% formic acid in 80% acetonitrile, followed by 80% acetonitrile	0.1~30.0	FF and FFA: 92~106 CIP, ENR and SAR: 85~107
Meat [15]	FF, FFA, CIP, ENR, SAR, and other analytes	HPLC–high resolution MS/MS	Extraction with 0.1 M EDTA and acetonitrile: water (8:2, *V*/*V*), followed by acetonitrile	FF and FFA: 10 CIP, ENR and SAR: 3.3	71~95
Pork, beef, and mutton [16]	FF, CIP, ENR, SAR, and other analytes	UHPLC–linear-ion-trap MS	Extraction with acetonitrile: water: 0.1% formic acid (80:19: 0.2, *V*/*V*/*V*), SPE clean-up with HLB cartridge	FF: 20 CIP, ENR and SAR: 2	FF: 84.5~94.1 CIP, ENR and SAR: 67.4~118.8
Fish [17]	FFA, ENR, SAR, and other analytes	HPLC–ion-trap MS	Extraction with acetonitrile	FFA: 0.1 ppm ENR and SAR: 0.01 ppm	>50
Frog legs and other aquacultured species [18]	FF, CIP, and ENR	HPLC–quadrupole-time of flight MS	Extraction with 1% acetic acid, acetonitrile, sodium chloride, and ceramic homogenizers	FF: 1.4 * CIP: 0.5 ENR: 0.8	80~130
Rainbow trout muscle [28]	FF, FFA, CIP, and ENR	HPLC–UVD	Extraction with ethyl acetate, SPE clean-up with C18 cartridge	17.7~39.1	71.1~94.7
Egg (This study)	FF, FFA, CIP, ENR, and SAR	HPLC–FLD	Extraction with 0.1 M EDTA disodium solution, water and acetonitrile, followed by a QuEChERS procedure	FF and FFA: 2.0~5.0 CIP, ENR and SAR: 0.1~0.2	71.9~94.8

Note: -, not provided. *, the values presented in this table represent the LODs.

**Table 4:** The test results of the established QuEChERS-HPLC-FLD method applied to real samples.

Sample	Brand	Source	Test Results (µg/kg)
1~6	free-range egg brand 1	wholesale market	Not detected
7~12	free-range egg brand 2	e-commerce platform	Not detected
13~19	feedlot industrial egg brand 1	wholesale market	Not detected
20~24	feedlot industrial egg brand 2	supermarket	Not detected
25~30	feedlot industrial egg brand 3	supermarket	Not detected
31~36	feedlot industrial egg brand 4	retail store	Not detected
37~42	feedlot industrial egg brand 5	e-commerce platform	Not detected

## Data Availability

All available data are contained within the article.

## Supplementary Materials

The following supporting information can be downloaded at: https://www.mdpi.com/1420-3049/29/1/252/s1. Table S1: The mean recoveries and precisions.
