# Supplementary material for: Development of a QuEChERS–HPLC–FLD Procedure for the Simultaneous Detection of Residues of Florfenicol, Its Metabolite Florfenicol Amine, and Three Fluoroquinolones in Eggs
Source: Molecules. 2024 Jan 3;29(1):252. doi: 10.3390/molecules29010252 (PMC10780869; doi:10.3390/molecules29010252)
Supplement: Supplementary file 1 [file molecules-29-00252-s001.zip › molecules-2770012-supplementary.pdf]

Table S1. The mean recoveries and precisions.

Matrix	Analyte	Fortified Level (μg/kg)	Recovery	Between-day RSD	Within-day RSD
Whole egg	FF	5.0	83.9	4.1	4.5
		10.0 *	93.3	3.4	3.4
		20.0	94.6	3.6	3.5
	FFA	2.0	71.9	4.5	4.9
		5.0	82.4	4.6	4.9
		10.0 *	81.6	4.2	5.1
		20.0	82.3	3.1	3.6
	CIP	0.1	76.0	4.3	4.9
		5.0	82.8	5.1	6.1
		10.0 *	83.6	3.5	3.6
		20.0	84.1	3.5	3.7
	ENR	0.1	87.1	5.1	6.2
		5.0	92.1	4.4	4.7
		10.0 *	94.0	4.2	3.9
		20.0	94.8	4.0	4.9
	SAR	0.2	82.0	4.9	5.2
		2.5	85.7	3.3	4.1
		5.0 *	87.3	3.9	4.6
		10.0	87.5	3.5	4.3
Egg yolk	FF	5.0	80.4	4.6	5.3
		10.0 *	87.5	4.0	3.8
		20.0	90.5	3.6	3.7
	FFA	2.0	73.2	4.3	4.7
		5.0	78.4	4.5	4.9
		10.0 *	81.4	4.7	4.6
		20.0	81.9	3.9	3.9
	CIP	0.1	72.6	4.6	5.8
		5.0	77.8	4.3	5.4
		10.0 *	82.2	4.6	5.3
		20.0	83.8	3.9	3.7
	ENR	0.1	86.2	6.6	7.3
		5.0	93.3	5.6	5.9
		10.0 *	93.9	4.2	4.4
		20.0	94.4	3.9	4.5
	SAR	0.2	73.5	5.1	5.1
		2.5	76.9	3.5	3.9
		5.0 *	79.4	4.2	4.8
		10.0	80.7	3.8	4.1
Egg albumen	FF	5.0	87.4	4.5	5.2
		10.0 *	90.9	4.2	4.3
		20.0	92.2	3.9	4.1
	FFA	2.0	75.1	4.9	5.8
		5.0	81.5	4.8	5.5
		10.0 *	80.8	4.4	5.6
		20.0	81.1	3.1	3.4
	CIP	0.1	75.3	4.2	4.6
		5.0	81.7	4.5	5.2
		10.0 *	82.2	3.4	4.8

	20.0	83.7	4.4	4.9
ENR	0.1	86.7	6.3	6.4
	5.0	93.1	5.7	6.3
	10.0 *	91.7	4.1	5.7
	20.0	94.6	3.9	4.6
SAR	0.2	82.4	6.1	7.0
	2.5	85.6	4.9	6.2
	5.0 *	88.2	4.1	4.8
	10.0	89.3	3.6	4.2

Note: *, MRL.
